# Cohesive Properties of Bimaterial Interfaces in Semiconductors: Experimental Study and Numerical Simulation Using an Inverse Cohesive Contact Approach

**DOI:** 10.3390/ma17020289

**Published:** 2024-01-06

**Authors:** Caio Adler, Pedro Morais, Alireza Akhavan-Safar, Ricardo J. C. Carbas, Eduardo A. S. Marques, Bala Karunamurthy, Lucas F. M. da Silva

**Affiliations:** 1 Faculdade de Engenharia, Universidade do Porto, Rua Dr. Roberto Frias, 4200-465 Porto, Portugal; linsadlercaio@gmail.com (C.A.); pedromoraisss99@gmail.com (P.M.); emarques@fe.up.pt (E.A.S.M.); lucas@fe.up.pt (L.F.M.d.S.); 2 Institute of Science and Innovation in Mechanical and Industrial Engineering (INEGI), Rua Dr. Roberto Frias, 4200-465 Porto, Portugal; 3 Infineon Technologies Austria AG, Siemensstrasse 2, 9500 Villach, Austria; bala.karunamurthy@infineon.com

**Keywords:** semiconductors, bimaterial interface, cohesive zone modeling, interfacial fracture, fracture energy

## Abstract

Examining crack propagation at the interface of bimaterial components under various conditions is essential for improving the reliability of semiconductor designs. However, the fracture behavior of bimaterial interfaces has been relatively underexplored in the literature, particularly in terms of numerical predictions. Numerical simulations offer vital insights into the evolution of interfacial damage and stress distribution in wafers, showcasing their dependence on material properties. The lack of knowledge about specific interfaces poses a significant obstacle to the development of new products and necessitates active remediation for further progress. The objective of this paper is twofold: firstly, to experimentally investigate the behavior of bimaterial interfaces commonly found in semiconductors under quasi-static loading conditions, and secondly, to determine their respective interfacial cohesive properties using an inverse cohesive zone modeling approach. For this purpose, double cantilever beam specimens were manufactured that allow Mode I static fracture analysis of the interfaces. A compliance-based method was used to obtain the crack size during the tests and the Mode I energy release rate ( $G_{Ic}$ ). Experimental results were utilized to simulate the behavior of different interfaces under specific test conditions in Abaqus. The simulation aimed to extract the interfacial cohesive contact properties of the studied bimaterial interfaces. These properties enable designers to predict the strength of the interfaces, particularly under Mode I loading conditions. To this extent, the cohesive zone modeling (CZM) assisted in defining the behavior of the damage propagation through the bimaterial interfaces. As a result, for the silicon–epoxy molding compound (EMC) interface, the results for maximum strength and $G_{Ic}$ are, respectively, 26 MPa and 0.05 N/mm. The second interface tested consisted of polyimide and silicon oxide between the silicon and EMC layers, and the results obtained are 21.5 MPa for the maximum tensile strength and 0.02 N/mm for $G_{Ic}$ . This study’s findings aid in predicting and mitigating failure modes in the studied chip packaging. The insights offer directions for future research, focusing on enhancing material properties and exploring the impact of manufacturing parameters and temperature conditions on delamination in multilayer semiconductors.

## 1. Introduction

As products get more complex, the interaction between different materials and parts becomes a big factor in making new ideas possible. Hence, it is essential to study the interfacial behavior of two different materials to ensure a more reliable design. In the context of the semiconductors, the interaction between the interfaces happens under a gradient of temperature and loads, which may inflict damage leading to an interfacial failure. Accordingly, to prevent inconsistencies, it is essential to have sufficient knowledge of the interfacial properties of bimaterial components. Some of the most common materials used in the semiconductor industry are silicons, epoxy molding compounds (EMCs), polyimides, and silicon oxide. Silicon is a fundamental material in semiconductor devices. Silicon is the primary material used to fabricate integrated circuits (ICs), which are the building blocks of electronic devices such as microprocessors, memory chips, and other digital and analog circuits. These ICs are essential for the functioning of computers, smartphones, and many other electronic devices. Silicon is also the foundation for creating transistors, the basic units of electronic switches. Transistors are employed for signal amplification, switching, and digital logic operations in electronic circuits. EMCs are widely used in packaging semiconductor devices, and their interaction with silicon is critical for the structural integrity of semiconductor components. Polyimide is another material used in semiconductor industries. It is often used as a dielectric material for insulating layers in semiconductor devices. Its high thermal stability, excellent chemical resistance, and electrical insulation properties make it suitable for creating thin films that isolate different layers of semiconductor components. Silicon oxide is a widely used buffer layer in the semiconductor industry. It is a dielectric material that provides excellent insulation between the substrate and the active layer. These materials represent interfaces commonly encountered in semiconductor manufacturing processes. Silicon, which is renowned for its favorable mechanical and electrical properties, is susceptible to brittle behavior. Consequently, materials like EMCs are extensively utilized to safeguard delicate components from moisture, heat, and shocks. Due to the great range of applications of silicon and EMCs, their properties have been extensively studied, but this does not apply to their interfaces when they are used together in a bimaterial component. Because of their behavior, a weak interface may surge between the two materials causing the beginning of a crack initiation and its subsequent propagation. However, there is still a lack of information on how it would propagate. As mentioned, delamination is a crucial challenge in power electronics packages, exerting a considerable influence on their reliability and performance. This impact is particularly pronounced due to the substantial thermomechanical loads generated by the latest devices handling large amounts of electrical power [1]. Analyzing potential weak points in multimaterial components involves calculating fracture energy using concepts from fracture mechanics [2]. The calculation contains many variables, so to obtain a deep understanding of the materials interfaces, it is important to define the loading modes. There are three main directions (or loading modes) that contribute to crack propagation: Mode I (tensile or opening mode), Mode II (shear or sliding mode), and Mode III (tearing or tearing mode). However, this study focuses on the first mode (Mode I or tensile), which stands for stress orthogonal to the crack plane of the crack propagation [3]. While the silicon–EMC interface has received limited analysis, other types of interfaces are more thoroughly explored and discussed in the literature. Fan et al. [4] investigated the mechanism of cohesion between EMC and copper through Atomic Force Microscopy (AFM). Khan et al. [5] described the adhesion between transferred graphene and silicon. As the results show, adhesion varies significantly with changes in the graphene used. The values found for the interface of silicon and graphene are very similar to the Mode I fracture energy ( $G_{Ic}$ ) found for the interface of EMC and copper, as Samet et al. [6] show. Wang et al. [7] assessed interfacial fracture toughness in flip-chip packages and bimaterial systems, using a six-axis submicron tester, thermal chamber, FEM modeling, and laser interferometry. The results show a value of 0.02 N/mm for the fracture toughness under Mode II loading conditions. Modified cohesive zone parameters for polymers were introduced by Kwatra et al. [8], showcasing the ability of the cohesive zone modeling (CZM) approach to capture load–displacement plots in microelectronic packages featuring an EMC on a copper leadframe. The mixed-mode fracture of EMC–copper was also analyzed by Krieger et al. [9] using the CZM approach. They introduced a framework for determining mixed-mode cohesive zone parameters through experimental methods. Raghavan et al. [10] developed a framework based on the cohesive zone modeling approach to investigate interfacial delamination in sub-micron-thick layers. Subsequently, this framework was applied to predict the reliability of microelectronic devices. None of the studies evaluate silicon–EMC interfaces under Mode I; due to this reason, this paper aims to study this specific situation, as well as the interface between an EMC and polyimide. Through the analyzed references, there are multiple ways to conduct the fracture test. Kim et al. [11] and Samet et al. [12] used double cantilever beams (DCBs). This is a similar approach to the one Samet et al. [6] used for copper and EMC interfaces. It is important to mention the influence of external factors on the results obtained through the tests. A change in temperature and humidity can influence the interfacial strength. A rise in either of the factors will contribute to a lower strength [8]. Another factor that can contribute to a better interfacial property is adding a very thin layer of another material. As indicated by Wang et al. [7], the addition of a new layer can induce a better interface contact and therefore a higher $G_{Ic}$ in the context of this study. The addition of new materials can also improve the optoelectrical, photovoltaic, and conductive properties of the combination of materials [13]. All these mentioned properties are important for the semiconductors industry. For this reason, the second interface type used has a layer of silicon oxide and one layer of polyimide between the silicon and EMC, which is referred to as Type II in the current study.

As mentioned above, numerical modeling can provide important data for the interfacial parameters of the studied materials. The two main methods used for delamination are the CZM and the Virtual Crack Closure Technique (VCCT) [12]. As observed by Cunha et al. [14], CZM requires a predefinition of the damage propagation. However, it also presents advantages compared with VCCT. Any initial flaw in VCCT will contribute to unstable crack propagation and severe convergence problems [15]. As the properties of the bimaterial interfaces considered for this study have not yet been thoroughly analyzed and obtained, a CZM method is preferred. The finite element (FE) modeling approach was employed by Mirone et al. [1] for delamination analysis in power electronics packages. Their study includes guidelines for calibration and utilizes a global–local approach with a bilinear cohesive zone model (CZM). Through simulations on a reference package, this study conducted a parametric study and sensitivity analysis, identifying crucial factors affecting delamination behavior. As indicated by Kwatra et al. [8], the maximum traction and initial stiffness for a Mode I loading EMC–copper interface are, respectively, 30 MPa and 126,422 MPa/mm. A similar study was conducted by Krieger et al. [9], where similar values for both parameters were found. In the context of semiconductors, Raghavan et al. [10] studied the cohesive properties of silicon and BEOL (BEOL stands for “back-end of line”, which consists of copper layers intersected by low-k dielectric materials), where they found 1.8 MPa and 10,000 MPa/mm for the maximum traction and initial stiffness, respectively. All the mentioned studies utilize cohesive interactions for interface modeling. However, Krieger et al. [9] opted to utilize VCCT in another analysis. There is still a lack of information about the silicon–EMC and EMC–polyimide interfaces, although the values found in the literature for similar interfaces are useful in setting values for comparison.

This paper aims to experimentally and numerically conduct Mode I fracture tests to determine the cohesive behavior of two types of interfaces. To obtain the fracture energy under Mode I, the compliance-based beam method (CBBM) [16] was considered. From the experimental results, an inverse cohesive contact approach was conducted for both interfaces, resulting in the values for the cohesive properties and stress and damage distribution through the tested wafers.

## 2. Materials and Methods

### 2.1. Materials and Geometry

**Figure 1:**
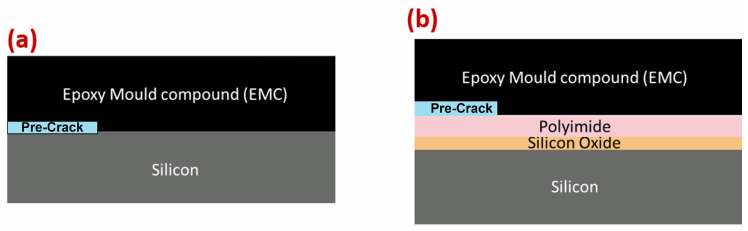
Schematics for the Type I interface (**a**) and Type II (**b**) interface. Dimensions in mm. (The scheme is not to scale).

There were two types of interfaces analyzed in this study: silicon–EMC (referred to as Type I, (Figure 1a)) and EMC–polyimide (referred to as Type II, (Figure 1b)). A layer of gold acting as the precrack was utilized. Figure 1 is a schematic that clarifies the layers’ disposition.

#### 2.1.1. Materials

Three main components were used in the experimental tests for the Type I interface: the wafer, which consists of silicon and EMC; steel bars to support the wafers during the test; and the adhesive that bonds the steel bars to the wafer. In addition, the Type II interface contains additional silicon oxide and polyimide layers between the silicon and EMC layers. To this extent, Table 1 summarizes the elastic properties of these materials. To manufacture the DCBs, an epoxy-based structural adhesive was used to bond the wafers to the steel supports. For the adhesive, it is important to ensure that it has enough strength to handle the test loads without failure.

The EMC used in the tested components consists of glass fiber-reinforced epoxy and may vary in terms of the fiber’s density, length, and composition according to the manufacturer. Due to a broad range of variables, the properties shown in Table 1 for the EMC are from the wafer’s manufacturer and reflect the actual EMC used. The other material used is silicon. In contrast with the EMC, it is a brittle material and presents a Mode I fracture energy of around 0.00173 N/mm [2,17]. The steel used is PM300, which presents a much higher ultimate tensile strength and Young’s Modulus compared with the other materials studied; accordingly, its deformation during the tests and numerical simulations is irrelevant.

In the context of the Type II interface, it is also important to define the properties of silicon oxide and polyimide. Polyimide presents a plastic behavior typical of thermoplastics, though it was considered elastic to simplify the simulation. The properties of silicon oxide and polyimide are shown in Table 1. As the objective of this study is to obtain an interfacial failure in the silicon–EMC interface, failure at the adhesive layer used to bond the wafer to the steel supports is unacceptable. To this extent, AV138 M1 combined with the HV 998–1 hardener was used as a high-strength epoxy-based adhesive. The properties of the adhesive are summarized in Table 2. As an epoxy, this adhesive is very stiff with high strength.

**Table 1:** List of material properties [18].

Materials	Ultimate Tensile Strength (MPa)	Poisson’s Ratio	Young’s Modulus (GPa)
Steel PM300	1020	0.33	205
Silicon	170	0.28	130
EMC	120	0.28	24
Siliocon Oxide	129	0.3	75
Polyimide	215	0.35	2.5

**Table 2:** Araldite AV138 adhesive (Huntsman, Switzerland) properties [19].

Young’s Modulus (GPa)	4.59
Tensile Yield Strength (MPa)	36.49
Ultimate Tensile Strength (MPa)	41.01
Tensile Failure Strain (%)	1.3
Poisson’s Ratio	0.35

#### 2.1.2. Geometry

**Figure 2:**
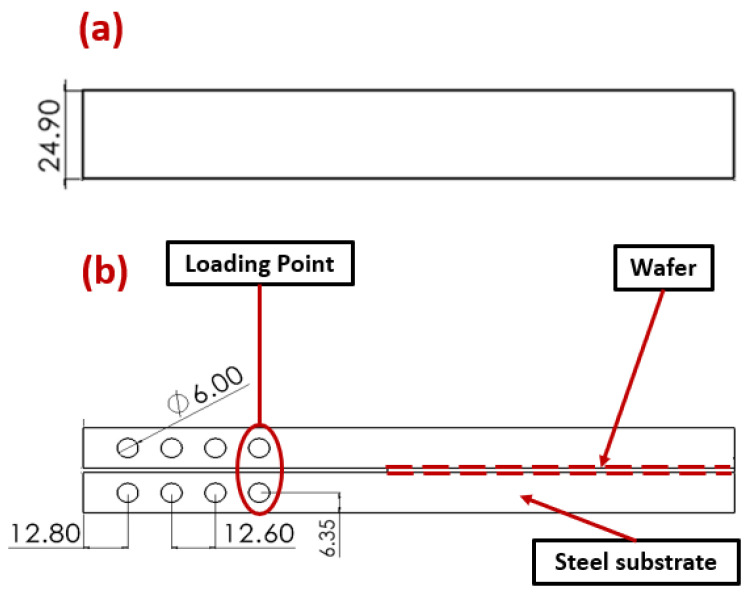
Top (**a**) and side (**b**) view of the DCB specimen. Dimensions in mm.

Figure 2 represents the technical drawing of the DCB joint and the wafer’s geometry. The DCB consists of two steel bars with the wafer in between (Figure 2a). The holes made on the steel bars are used to apply the load to the wafers through loading pins. Due to the small thickness of the polyimide and silicon oxide layer, the technical drawings for both interfaces are the same (Figure 1).

The selection of element size, specifically thickness, was determined in alignment with the real-world application specified by the industry. However, the width and length were chosen to ensure compatibility with double cantilever beam (DCB) testing in universal testing machines.

### 2.2. Manufacturing Procedure

**Figure 3:**
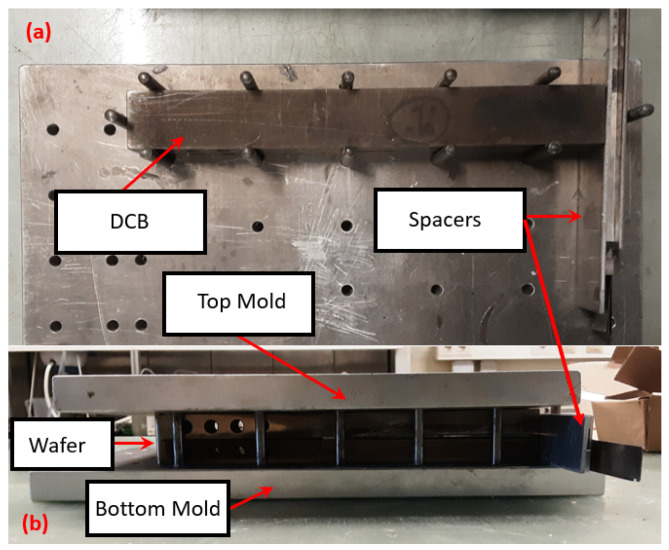
Different views of the DCB joint in the mold: (**a**) top view and side view (**b**) curing at room temperature with controlled pressure.

The manufacturing process of the DCB joints starts by sandblasting the surface of the steel bar. Sandblasting increases the surface roughness and removes corrosion and contamination, which are important factors for good adhesion. After sandblasting, it is crucial to remove any spare sand on the surface with compressed air and then clean the bar with acetone. The preparation of the wafer starts by applying 800-grit sandpaper on both sides at $45^{\circ }$ , then the surfaces were cleaned with acetone followed by 6 s of plasma treatment. From test data, this process is known to increase the EMC’s surface energy by around 199%. A more mild increase can also be measured for the silicon surface, as the surface energy goes from 30.51 mJ/m^2^ to 39.94 mJ/m^2^ after the treatment. After preparing the bar and the wafer, it is necessary to use a mold in order to control the alignment of the DCBs and the applied pressure during the curing process. The mold consists of two aluminum plates with small holes made for inserting pins that hold the DCBs in place (Figure 3). However, due to the length of the steel bars used in the DCB, it is necessary to place spacers behind it so the bar is tightly placed on the mold. Before applying the adhesive on the treated surface, it is necessary to mix 5 g of the adhesive AV138 M1 for every 2 g of HV988-1 hardener. The curing proceeds under controlled pressure, applied on the mold, at room temperature for 24 h and then post curing at 60 °C for 2 h.

### 2.3. Testing Procedure

**Figure 4:**
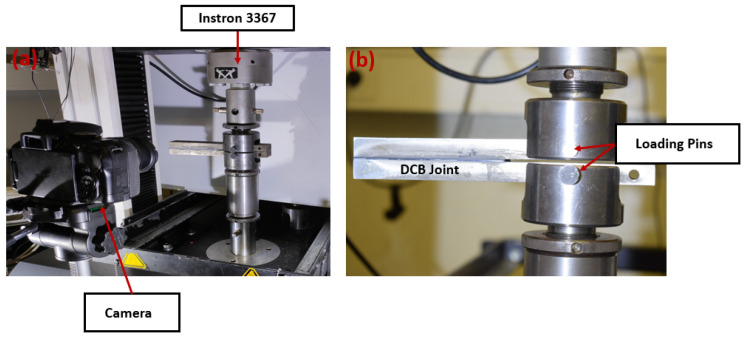
DCB under quasi-static loading condition: view of the testing machine (**a**) and DCB under quasi-static load (**b**).

The load was applied through the hole nearest to the wafer under room conditions, always in Mode I. The quasi-static test was conducted using a hydraulic test machine (INSTRON 3367, USA) under a constant displacement rate of 0.2 mm/min (Figure 4).

The data from the machine was later treated using a Compliance-Based Beam Method (CBBM) [16] (Equation (1)). This method allows us to determine the energy release rate of the crack without requiring the direct measurement of the crack size during testing. Instead, an equivalent crack length ( $a_{eq}$ ) is calculated from the specimen’s compliance (*C*) (Equation (3)) and then used in Equation (1). The data are used to determine the critical fracture energy, and it is later applied in Equation (1), where *G* is the modulus of the specimen, $E_{f}$ is the flexural modulus (obtained from Equation (2)), *P* is the load, *h* is the thickness of the substrate, and *B* is the width of the specimen [16].

(1) $$G_{Ic}=\frac{6P^{2}}{B^{2}h}\left(\frac{2a_{eq}^{2}}{h^{2}E_{f}}+\frac{1}{5G}\right)$$

(2) $$E_{f}={\left(C_{0}-\frac{12(a_{0}+|\Delta |)}{5Gbh}+\frac{1}{5G}\right)}^{-1}\frac{8(a_{0}+{\left|\Delta |\right)}^{3}}{bh^{3}}$$

(3) $$C=\frac{\delta }{P}=\frac{8a_{eq}^{3}}{bh^{3}E_{f}}+\frac{12a_{eq}}{5Gbh}$$

The flexural modulus represents the stress concentration around the crack tip and is affected by the substrate dimensions (*h* and *b*), the initial crack length ( $a_{0}$ ), the adherent shear modulus (*G*), and the initial compliance ( $C_{0}$ ). *C* in Equation (3) is calculated by the ratio of displacement ( δ ) to the applied load (*P*). A correction factor for the crack length ( Δ ) is applied and determined by the following equation:

(4) $$\begin{array}{cc} \Delta & =h\sqrt{\frac{E{\left(3-2\frac{\tau }{1+\tau }\right)}^{2}}{11G}} \end{array}$$

(5) $$\begin{array}{cc} \tau & =\frac{1.18E}{G} \end{array}$$

where *E* is the Young’s Modulus of the substrate (steel bars in this study).

### 2.4. Numerical Modeling

#### 2.4.1. Geometry

**Figure 5:**
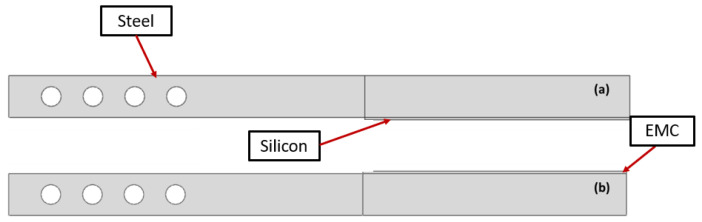
Top part (**a**) and bottom part (**b**) used in the numerical simulation for Type I interface.

The geometry of the Type I interface can be seen in Figure 5. The Type I interface geometry was created in two parts. The top part, represented in Figure 5a, consists of a steel bar and silicon, and the other part (Figure 5b) of the EMC and a steel bar. A cohesive interaction was defined between the EMC and silicon surfaces. For Type II, the top half also includes a layer of silicon oxide and a layer of polyimide. These additional thin layers were added as different parts to apply a more refined mesh.

The quasi-static analysis was defined as the simulation type. Hence, a displacement control quasi-static test was conducted The history output permits to measurement of localized parameters, such as displacements and reaction forces in specific reference points or regions. Thus, the reaction force was measured at RP2 and the displacement at RP1 as discussed later in Section 2.4.5. In contrast, the field output permits measurement data to be distributed throughout the entire model. Parameters such as stress and damage were measured by this method.

From the created geometry, it is necessary to apply interactions between the created parts for an accurate simulation of the behavior of the DCB. From the experimental test, it is known that the crack will propagate between silicon and EMC for the Type I interface and between polyimide and the EMC for Type II. Thus, the damage path was defined by a cohesive surface model along these interfaces. This type of interaction requires three main parameters to be fully specified: fracture energy, initial stiffness, and maximum traction. The fracture energy values for both interfaces were obtained experimentally. The other two parameters were obtained from an inverse cohesive contact approach and can be seen in the Section 3 and Section 4.

#### 2.4.2. Cohesive Behavior

**Figure 6:**
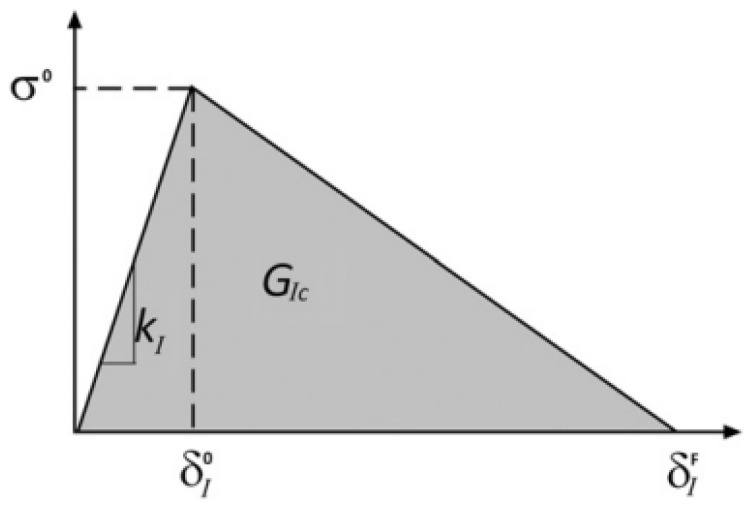
Traction separation law considered in this study.

For the experimental values obtained, it is essential to define a model for the damage propagation. The behavior of the interaction between silicon and the EMC was dictated by the stiffness coefficients, maximum traction, and fracture energy of the interfaces. As the value of $G_{Ic}$ was known, an inverse approach was used in order to define the stiffness and maximum traction. It consisted of applying values for the two parameters and then analyzing the load–displacement curve created from the analysis. The final values for maximum traction and initial stiffness are obtained when the experimental and numerical load–displacement curves match. All these parameters are important for a CZM analysis, which has been widely used for modeling crack propagation through interfaces [20]. This method assumes the existence of a cohesive surface, where the damage will initiate and propagate [21]. The damage is represented by a traction–separation curve, where the area under the curve is the fracture energy for the analyzed loading mode (Figure 6). The slope of the initial part ( $\delta <\delta_{I}^{0}$ ) is the normal cohesive stiffness. $\sigma^{0}$ is the maximum traction in this model. After $\delta_{I}^{0}$ is reached, the damage starts and will increase until it reaches 1 at $\delta_{I}^{F}$ . At this juncture, the stiffness is zero, signifying the absence of interactions between the surfaces in contact.

#### 2.4.3. Damage Evolution

**Figure 7:**
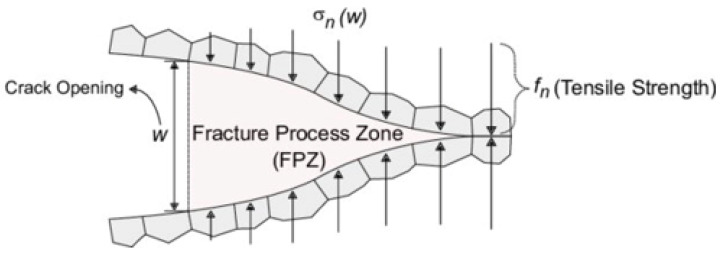
Schematics of the damage propagation through the interface [22].

The damage propagates through a gradual process following the bimaterial interface. Each point along the interface experiences a different stage of damage (Figure 7). As shown in Figure 7 the damage at the crack tip is more pronounced, with higher values. Conversely, for material points farther from this location, the damage level diminishes and even reaches zero. The damage index ranges from 0 (indicating no damage) for points distant from the crack tip to 1 (representing complete element failure) for the crack tip itself. The arrows in Figure 7 illustrate the traction applied to the crack surfaces. The traction is minimal for points in proximity to the crack tip and increases with distance from the crack tip.

#### 2.4.4. Mesh

**Figure 8:**
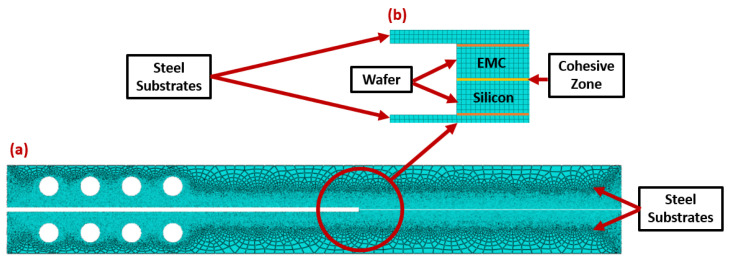
Mesh applied for the Type II interface assembly (**a**) and zoom of the cohesive zone (**b**).

For the Type I interface, a global mesh with a medium density of 1 mm was applied to the entire model. Because of the thin films used in the Type II interface, a more refined mesh on the wafer’s zone was required. For this extent, a local mesh with an approximate element size of 0.08 mm was applied (Figure 8). All the mesh elements types are considered as plane stress.

#### 2.4.5. Boundary Conditions

**Figure 9:**
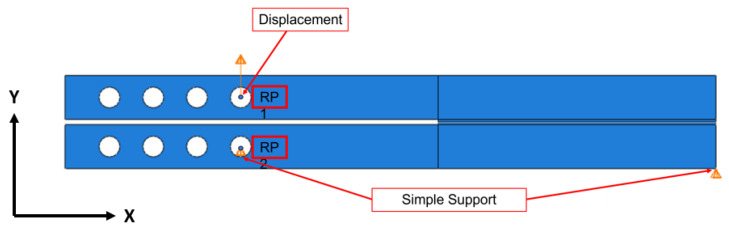
Boundary conditions for both interfaces’ analysis.

The boundary conditions for both interfaces were derived from the experimental test. The top steel bar experienced a 1 mm displacement at reference point 1 (RP1). The bottom bar was constrained similarly to a pin, prohibiting movement along the y-direction and rotations. These conditions restrict the model’s y-directional movement and bottom bar rotations (Figure 9).

The reference points are also crucial for measuring the load and displacement that the DCB is subjected to during the simulation. The steps module in Abaqus permits defining the measured parameters during a simulation.

## 3. Results

From the numerical simulation, the direct output is the load–displacement curve obtained from the input data (experimental results). The simulation tried to match the curves obtained with the experimental load–displacement curves by manipulating the values for initial stiffness and maximum traction. The material properties used for the simulation are shown in Table 2.

### 3.1. Experimental Results

**Figure 10:**
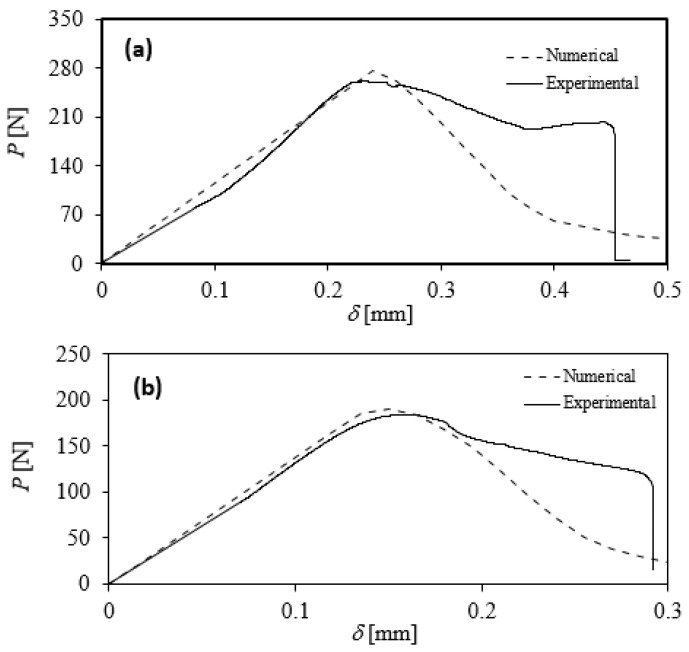
Comparison of numerical and experimental load–displacement curves for Type I interface (**a**) and Type II interface (**b**).

**Figure 11:**
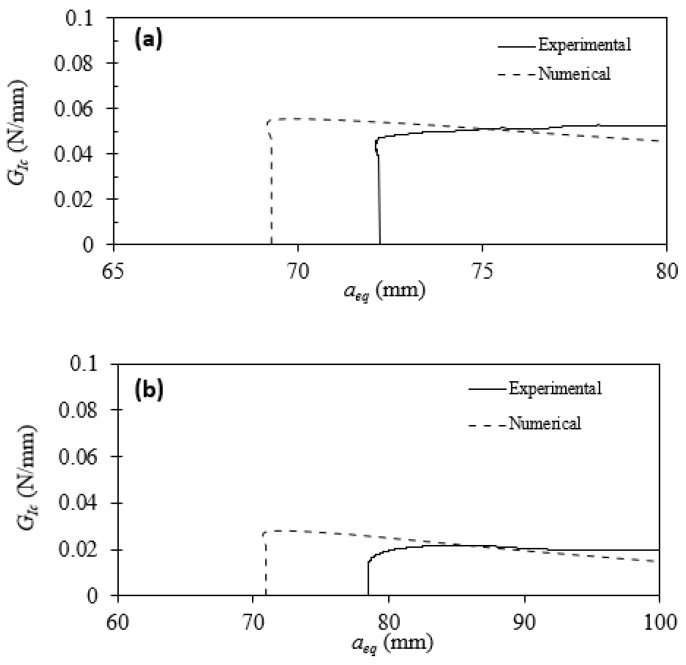
Comparison of numerical and experimental R-curves for Type I interface (**a**) and Type II interface (**b**).

The quasi-static results for Type I are shown in Figure 10a. Using the CBBM approach, the resistance curve (R curve) was also obtained, and it represents the energy release rate for pure Mode I ( $G_{Ic}$ ) as a function of equivalent crack length (Figure 11a). The same analysis was conducted for the Type II interface (Figure 10b and Figure 11b). The numerical results shown in Figure 10 and Figure 11 are discussed later in Section 4. In the Mode I DCB fracture test, the load required for increasing damage/crack size indeed displays non-monotonic behavior. During Mode I DCB fracture tests, as damage initiates at the precrack tip, the load necessary for enlarging the damage/crack size decreases with displacement. This reduction in load can vary based on the type of material/interface, exhibiting either a ductile response or a brittle behavior. Specifically, the reduction in load may occur with a lower or higher slope, respectively, depending on the material/interface characteristics. For the tested configurations, after damage initiation, the initial crack propagation at the beginning of the test shows a relatively abrupt reduction in load in both tested configurations. In the tested specimens, this initial crack propagation appears to be relatively unstable in the beginning, but as displacement increases, a more stable crack propagation is observed.

**Figure 12:**
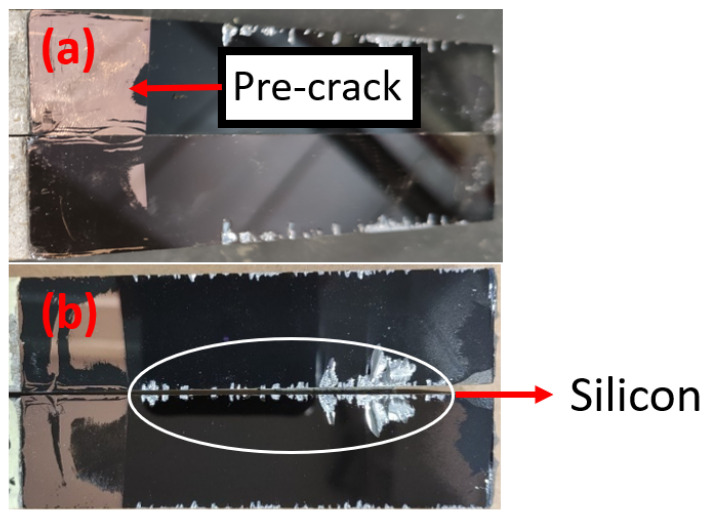
Type I (**a**) and Type II (**b**) interfaces.

For the Type I interface, it is possible to compare the obtained values with the results obtained by Samet et al. [12]. Despite using a different material along with silicon, the $G_{Ic}$ obtained was very similar. A small scatter in the results was obtained as the $G_{Ic}$ ranged from 0.050 to 0.052. The values obtained for the Type II interface are also very similar if compared with the literature [12]. However, the data are more scattered compared with the Type I interface ( $G_{Ic}$ = 0.0425 ± 52%). Due to the significant variation in the results obtained for Type II, one of the experimental results (as shown in Figure 10) was considered for the numerical analysis (accordingly, the $G_{Ic}$ = 0.02 was used in the numerical simulations). Further evaluation of the results can be performed by analyzing the path of the crack propagation. Many factors might contribute to a different path for the crack and the scattering of the results, as shown in Figure 10 and Figure 11. Any unintended damage propagation in the manufacturing phase may cause a deviation in crack propagation during the tests. Figure 12 shows the joint after the tests and full separation of the interfaces. The damage starts propagating from the pre-crack and develops along the wafer’s length. It is noticeable that the damage path tends to be different on the borders of the wafer compared with the center. The silver part shown in Figure 12 is silicon, which means that the crack deviated from the interface at the edges of the specimen.

As presented in the literature, the scatter of results is a common phenomenon, mainly due to the brittle nature of the silicon and also the multiple thin layers that exist in Type II specimens. Values for $G_{Ic}$ in copper–EMC interfaces vary from 0.036 to 0.060 N/mm [8,9,23,24]. Kwatra et al. [8] studied the thermal aging effects on EMC–copper leadframe interfacial adhesion in microelectronic packages using cohesive zone models. Krieger et al. [9] introduced a framework using cohesive zone theory for crack propagation modeling without pre-existing cracks. The method is demonstrated on a copper/epoxy interface, showcasing the determination of cohesive zone parameters through experimental methods. The molding compound in power modules was explored by Calabretta et al. [23], focusing on copper–resin interface adhesion, using tests, finite element analysis, and cohesive elements for validation. The fracture energy of the copper–EMC interface was also analyzed by Rambhatla [24] using a new test setup called Crowbar Loading (CBL). The results were compared with DCB samples.

### 3.2. Numerical Results

#### Interfacial Properties

Through an iterative process using an inverse CZM approach, the cohesive values were adjusted until the numerical load–displacement curves aligned with the experimental data in terms of initial slope and maximum load. Subsequently, similar to the experimental part, the CBBM was employed to determine the numerical $G_{Ic}$ value. The results are shown in Table 3. The scatter in the results is quite prominent for the Type II interface. This is primarily attributed to potential damage in the silicon layer before testing and the presence of multiple very thin films (layers) in this type of interface. In the numerical simulation, as expected, the failure happened between the silicon and EMC for the Type I interface and between the polyimide and EMC for the Type II interface. The parameter that describes damage in this simulation is the CSDMG, which represents the damage that the joint suffered, ranging from 0 to 1, where 1 is full separation (complete failure). Table 3 presents the comparison between the normal interfacial strength and the stiffness of the interfaces under study compared with the data reported in the literature. The difference in values for initial stiffness between the numerical simulation and the literature is notable. This discrepancy is a result of the inverse approach used in the numerical simulation. The numerical input came from experimental data, which are subjected to a much higher displacement. This displacement is caused by the compliance of the test machine, but the reaction force measured during the test is still accurate, which led to a maximum traction much closer to that observed in the literature.

## 4. Discussion

The obtained experimental and numerical load–displacement curves and R-curves were compared. They are similar in initial slope and maximum load, though their format is still different in the damage evolution part (Figure 10). The triangular shape is obtained from the power law selected for numerical analysis. The power law dictates the evolution of the traction–separation curves. Based on the experimental data, the power law could be adapted to a trapezoidal law to recreate the plateau seen in some of the experimental tests. However, this plateau will not influence the use of this model for a safe life design. This numerical model can simulate the damage initiation and the maximum strength of the interface, two of the main parameters considered for safety purposes. This plateau will influence the R-curve as well. As observed in Figure 11, the decline in value for the numerical $G_{Ic}$ happens after its peak. The explanation for this phenomenon resides in the numerical load–displacement curve format. In contrast with the experimental load–displacement curve, the numerical curve presents the steepest decline in maximum load after its peak. This results in a decline in the numerical $G_{Ic}$ , as both curves are related. Another difference between the numerical and experimental results observed in Figure 11 is the initial crack length. This is caused by a lower numerical compliance. Due to imperfections in the experimental data, the compliance value increases to compensate, causing the R-curve to shift leftward.

### 4.1. Damage Evolution

**Figure 13:**
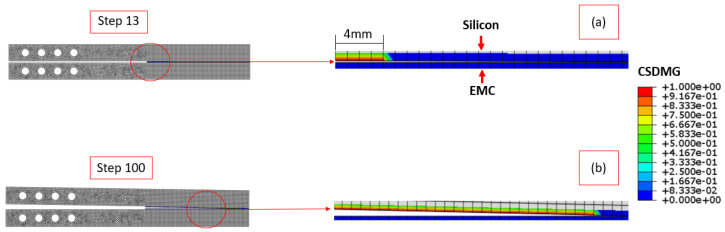
Damage distribution at steps 13 (**a**) and 100 (**b**) for Type I interface.

For the Type I interface, the cohesion rupture initially happens at step 13 (out of 100). From this step, the damage propagates through the interface until the simulation ends or the two interfaces are fully separated. The parameter that measures damage is the “CSDMG”. This parameter considers that the damage is characterized by progressive degradation of the material stiffness and uses mesh-independent measures to drive the damage evolution. The Type I interface damage distribution can be seen in Figure 13, where Figure 13a represents step 13 and Figure 13b the results for step 100.

**Figure 14:**
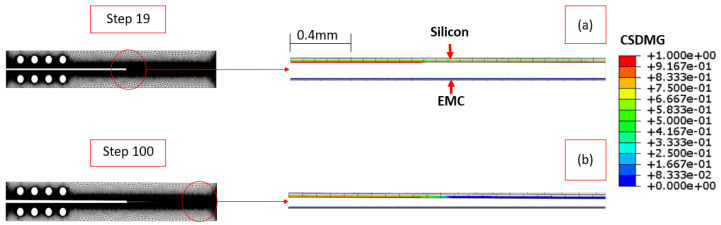
Damage distribution at steps 19 (**a**) and 100 (**b**) for Type II interface.

A similar analysis was applied to the Type II interface. The initial rupture occurs at step 19, with a higher displacement than the Type I interface (Figure 14a). At the final step (100), both interfaces are not fully separated (Figure 14b). The more refined mesh makes visualization challenging, necessitating a closer zoom at the start (Figure 14a) and end (Figure 14b) of the wafer.

### 4.2. Stress Distribution

**Figure 15:**
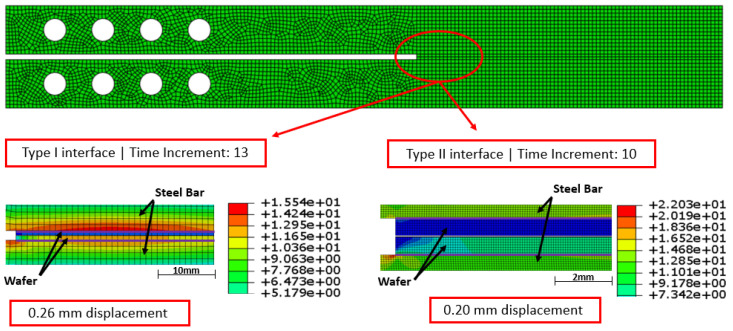
von Mises stress distribution at the most critical zone for Types I and II DCB (values in MPa).

From the obtained simulation, it is possible to evaluate if the stress in any section of the model surpassed the material’s ultimate tensile strength. This is a parameter that indicates the ultimate tension that the material can support before rupture. As indicated in Figure 15, in both simulations, the maximum von Mises stress was under the values for ultimate tensile strength indicated in Table 1. This analysis indicates that the CZM is applicable for the studied joints, as none of the materials studied suffered from a rupture under the test conditions used.

In conclusion, the values for initial stiffness and maximum traction obtained were compared with the literature. The difference in values for the initial stiffness is clear, which might indicate an inaccuracy in the simulation. Nevertheless, the maximum traction obtained is in line with the literature and should not be disregarded. For the load–displacement curves obtained, the Type I interface presents a very similar result to the experimental data. For both interfaces to better simulate the damage evolution part, using a trapezoidal CZM law is suggested.

As discussed earlier in the Introduction section, the significance of studying multilayer configurations in microchip packages is well-known and has been explored in the literature. The mechanical response and cohesive damage properties of the bimaterial interfaces investigated in this paper contribute to understanding failure mechanisms in these complex structures. The results obtained help provide a better understanding of the failure mechanisms of bimaterial interfaces found in chip packaging. By considering the cohesive properties of each bimaterial interface, this paper aims to provide a foundation for predicting and mitigating potential failure modes in real-world applications. For future studies, modifications in manufacturing parameters (such as deposition temperature or EMC composition) can be considered to improve the interfacial strength and cohesive properties of the tested materials. Improving material properties to enhance their mechanical response (as explored, for example, in [25]) and enhancing their interfaces with other materials used in chip packages are also other aspects that can be explored in the future. Also, exploring the mechanical response of multilayer semiconductors under different temperature conditions to address a critical aspect of real-world applications is considered future work as well.

## 5. Conclusions

Although the properties of silicon and EMCs are well-known in the semiconductor industry, there exists a gap in understanding their interface behavior. To address this, the energy release rate under Mode I ( $G_{Ic}$ ) for these interfaces was investigated under quasi-static loading in this paper. This represents an important parameter to be considered when developing new products. Accordingly, EMC and silicon (Type I) and EMC, silicon, silicon oxide, and polyimide (Type II) wafers were analyzed. For the Type I interface, the value for $G_{Ic}$ was obtained as 0.051 ± 2%, while for Type II, the results show a fracture energy of 0.04 ± 52%. The presence of multiple layers in the Type II interface introduces a source of result variability. Notably, a key concern arises from microcracks in the silicon, occurring during wafer assembly handling or manufacturing. This information provides valuable insights for designing wafers, as delamination is a significant concern in these components. Numerical modeling for both interfaces was conducted by employing an inverse cohesive zone modeling approach. The numerical study allowed us to determine the cohesive properties of the bimaterial interfaces under investigation. This information can then be utilized to simulate and predict the interfacial behavior of similar bimaterial interfaces found in semiconductor wafers. From the numerical study, the initial stiffness and maximum traction were obtained. The values for the Type I interface are, respectively, 340 MPa/mm and 26 MPa, and for the Type II interface, they are 470 MPa/mm and 21.5 MPa. The stress analysis during crack propagation through the interface revealed that none of the analyzed materials experienced rupture, indicating that the crack propagated through the interface in both types.

The findings from this study contribute to predicting and mitigating failure modes in chip packaging with the specific configuration studied. These results provide insights for future research to improve material properties and examine the influence of manufacturing parameters and temperature conditions on delamination in multilayer semiconductors.

## Figures and Tables

**Table 3:** Table for comparing the different interfacial properties.

Interface	Initial Stiffness (MPa/mm)	Maximum Traction (MPa)	Ref.
Type I	340	26	-
Type II	470	21.5	-
Copper + EMC	95,978	31	[9]
Silicon + BEOL	10,000	1.8	[10]
Copper + EMC	126,422	30	[8]

## Data Availability

Data are included in the article.
